# Choriocapillaris flow deficits and choroidal vascular index in the fellow eye of patients with central serous chorioretinopathy

**DOI:** 10.1007/s00417-026-07147-x

**Published:** 2026-02-13

**Authors:** Leandro Inferrera, Dario Marangoni, Antonio Valastro, Marianna Presotto, Daniele Tognetto

**Affiliations:** 1 Department of Medical, Surgical Sciences and Health, University of Trieste, Trieste, Italy; 2 Department of Ophthalmology, Ospedale Beauregard, Azienda USL della Valle d’Aosta, Aosta, Italy

**Keywords:** Central serous chorioretinopathy, Angio-OCT, Choroidal vascular index, Flow deficit, Subfoveal choroidal thickness

## Abstract

**Purpose:**

To evaluate choroidal changes in the fellow eyes of patients with chronic central serous chorioretinopathy (CSCR).

**Methods:**

A retrospective study was conducted on 50 participants, including 25 patients with unilateral chronic CSCR and 25 age-matched healthy controls. Optical coherence tomography (OCT) enhanced depth imaging (EDI) and OCT-angiography (OCT-A) images were processed using ImageJ, and three parameters were analyzed: flow deficit (FD), subfoveal choroidal thickness (SFCT), and choroidal vascular index (CVI).

**Results:**

CSCR eyes had significantly higher SFCT (320.9 ± 59.5), FD (54.2 ± 9.2), and CVI (52.8 ± 18.2) compared to healthy controls (SFCT 263.8 ± 66.6; FD 43.9 ± 9.9; CVI 22.6 ± 11.8). No significant differences were found between CSCR and fellow eyes, but fellow eyes showed higher values (SFCT 296.6 ± 51.9; FD 44.5 ± 6.3; CVI 46.5 ± 20.3) than healthy controls. ANOVA confirmed group differences, with post-hoc tests showing CSCR eyes differed significantly from healthy controls. Pearson’s analysis found positive correlations for SFCT but inconsistent correlations between FD and CVI, limiting reliability.

**Conclusions:**

This study confirms choroidal vascular changes in both CSCR and fellow eyes, suggesting their role in pathogenesis and a possible genetic or environmental predisposition. These findings may help improve diagnosis, prognosis, and risk assessment in patients with CSCR.

## Key Messages

*****What is known***:**

- Central serous chorioretinopathy (CSC) is associated with choroidal vascular alterations, including increased subfoveal choroidal thickness and changes in choroidal vascularity.
- Previous studies suggest that subclinical choroidal abnormalities may also be present in the fellow eye of patients with unilateral CSC.

*****What is new***:**

- This study shows that flow deficit, choroidal vascularity index, and subfoveal choroidal thickness are altered in CSC eyes and exhibit measurable changes in clinically unaffected fellow eyes.
- Fellow eyes show intermediate choroidal changes compared to CSC eyes and healthy controls, supporting a physiological characterization of the well-recognized bilateral involvement in CSC.
- The combined assessment of Optical coherence tomography (OCT) and OCT-angiography derived parameters reveals measurable choroidal differences between CSC eyes, fellow eyes, and healthy controls.

## Introduction

Central serous chorioretinopathy (CSC) is a retinal condition characterized by a neurosensory retinal detachment [1]. Generally, it is a self-limiting pathology with a good visual acuity prognosis. However, in cases of chronic progression with persistent serous detachment, which leads to alterations in the retinal pigment epithelium (RPE) and photoreceptors, a reduction in visual acuity may occur [2, 3]. Imaging studies such as indocyanine green angiography (ICGA) have demonstrated that, in CSC, the choriocapillaris (CC) microvascular network is associated with multifocal areas of choroidal vascular hyperpermeability [4]. However, the exact pathogenesis of CSC remains under investigation. CC is the capillary network of the choroid, located beneath the retinal pigment epithelium (RPE). This structure is made up of a tightly packed array of blood vessels, crucial for supplying metabolic support to the RPE and photoreceptors [5, 6], delivering oxygen and nutrients while facilitating the removal of metabolic waste products.

The advent of non-invasive imaging techniques such as optical coherence tomography angiography (OCT-A) and enhanced depth imaging optical coherence tomography (EDI-OCT) has enabled detailed in vivo evaluation of the retinal and choriocapillaris vascular networks, as well as improved structural assessment of the choroid [7–9].

In parallel, the widespread availability of commercial swept-source and spectral-domain OCT (SD-OCT) devices has significantly enhanced image quality, providing greater signal penetration below the retinal pigment epithelium and thereby improving visualization of deeper choroidal structures [10, 11].

Compared with dye-based angiography, OCT-A allows the evaluation of the three-dimensional organization of the retinal and choriocapillaris circulation [12, 13]. The strong contrast achieved with OCTA allows for easy quantification using simple binarization methods, where pixels are categorized as either showing flow or no flow. This method facilitates the calculation of several parameters, such as vessel density and flow deficit (FD) [14, 15]. A typical en face OCTA image of the CC appears grainy, with alternating white and black pixels, making capillary details hard to see. This is attributed to OCTA’s resolution limits, noise, and the dynamic nature of CC circulation, which affects the consistency of FD measurements over time [6].

Quantitative evaluations of this “CC-like” structure have offered valuable insights into normal aging and various macular diseases [6, 15–22]. Several research groups have used various settings, such as axial slab positions, reference offsets, and thresholding methods. Since no universally accepted ground-truth validation standard exists, it’s challenging to claim one method is better than another. However, Byon and collaborators proposed that a 10-µm slab positioned 21–31 μm below the centerline of the retinal pigment epithelial band produced the most repeatable results [23].

Although OCT-A is essential for examining the retinal and choriocapillaris vascular networks, EDI-OCT remains the most effective method for evaluating choroidal structure and morphological changes, thereby facilitating the development of new parameters. Among these, one of the most widely used is the choroidal vascularity index (CVI) [9]. Several studies have demonstrated that this parameter is less affected by physiological changes, making it a valuable biomarker in both healthy and diseased eyes [24–27]. CVI has therefore emerged as a valuable tool for investigating the mechanisms of CSC, although recent evidence highlights that this parameter may be influenced by systemic and ocular conditions, underscoring the need for cautious interpretation [28].

While some studies have reported increased CVI in CSC and fellow eyes, others have found comparable values among CSC eyes, fellow eyes, and healthy controls [29, 30]. In this context, subfoveal choroidal thickness (SFCT) remains one of the most widely used quantitative parameters for evaluating choroidal involvement in CSC. Measured as the distance between the outer border of the retinal pigment epithelium and the choroidal–scleral interface, SFCT reflects overall choroidal thickening and has been consistently reported to be increased in eyes with CSC [31]. Increased SFCT is considered a hallmark of the pachychoroid phenotype and has been associated with choroidal vascular congestion and dilation of large choroidal vessels [35]. Importantly, several studies have also demonstrated increased SFCT in fellow eyes of patients with unilateral CSC, supporting the concept of bilateral choroidal predisposition [32].

The aim of this study was to identify alterations in microvascular perfusion in patients with chronic unilateral CSC by evaluating flow deficit (FD), choroidal vascularity index (CVI), and subfoveal choroidal thickness (SFCT), as well as examining the correlations among these parameters.

## Materials and methods

This retrospective study included patients with unilateral chronic CSC and a control group with healthy eyes, all recruited from the Ophthalmology Clinic of the Ospedale Maggiore in Trieste, Italy. Data were collected between February 2024 and December 2024. The study adhered to the principles of the Declaration of Helsinki. Informed consent was obtained from all individual participants included in the study.

Chronic CSC was defined based on a disease duration longer than 6 months and the presence of persistent or recurrent subretinal fluid documented on SD-OCT. The diagnosis was further supported by multimodal imaging findings obtained at the initial presentation of the disease, including fluorescein angiography (FA) showing characteristic leakage patterns and indocyanine green angiography (ICGA) demonstrating areas of choroidal hyperpermeability [4].

These imaging modalities were used at the initial presentation to support the diagnosis of CSC and to exclude other retinal or choroidal disorders, in accordance with standard clinical practice; they were not employed to define disease chronicity or for quantitative analysis [1].

Inclusion criteria included: Caucasian subjects aged 30 to 55 years with a diagnosis of unilateral chronic CSC.

Exclusion criteria included acute CSC, other retinal or vitreous disorders, prior photodynamic therapy, and systemic diseases affecting choroidal vascularization. Medical history was recorded for all patients, and a comprehensive ophthalmological examination was performed, including slit-lamp examination, best-corrected visual acuity (BCVA), tonometry, optical coherence tomography (OCT) with Enhanced Depth Imaging (EDI), OCT-angiography (OCTA), FA, and ICGA.

BCVA was collected as part of the routine clinical assessment to characterize the study population but was not included among the study outcomes and was not used for statistical analysis.

OCT scans were obtained using the HRA + OCT Spectralis device (Heidelberg Engineering GmbH, Heidelberg, Germany) with image processing performed using the integrated software, Heidelberg Eye Explorer (HEYEX), version 1.10.4.0 (Heidelberg Engineering GmbH, Germany). Image acquisition was conducted by two experienced examiners (LI and MP).

Clinical data for the study were extracted from the patients’ digital medical records and compiled into a database. For each patient, collected data included age, gender, date of CSC diagnosis, affected eye, subfoveal choroidal thickness (SFCT), choroidal vascularity index (CVI), and flow deficit (FD).

### OCTA imaging

Images were obtained using the HRA + OCT Spectralis device, with A-scans at a speed of 40 kHz and a scanning depth of 1.9 mm in tissue, providing an axial optical resolution of 7 μm and a lateral resolution of 14 μm.

Image acquisition was assisted by active eye tracking to automatically center the desired area. A single macular scan was acquired for each eye of each patient, centered on a 3 × 3 mm area. Scans were acquired in high-resolution (HR) mode with the following parameters: 1,536 A-scans, 512 B-scans, a 15°x15° scanning angle, a digital image size of 768 × 768 pixels, a scan time of 96 ms per image, a lateral resolution of 5 μm/pixel, an image acquisition rate of 9 Hz, and a maximum scanning depth of 8 mm.

Automated segmentation of the choriocapillaris (CC) was performed using the HEYEX software (20 μm thickness with a 10 μm depth from Bruch’s membrane to the choroid).

CC slab segmentation was visually inspected by three experienced graders (LI, DM and DT) to confirm correct positioning relative to Bruch’s membrane. Scans showing segmentation errors that could not be corrected through manual adjustment within the HEYEX software were excluded from further analysis. Only eyes with adequate segmentation were included, inappropriate segmentations were discarded.

Prior to image analysis, a contrast of 1:4 was applied uniformly across all images. Additionally, an automatic artifact removal algorithm was employed to eliminate flow signals from retinal vessels, ensuring that overlying retinal vessels did not affect the CC evaluation. The automatic artifact removal algorithm used to eliminate projection artifacts from overlying retinal vessels was the manufacturer-provided tool integrated into the HEYEX software. No custom-built or third-party artifact removal algorithms were applied. Only images with a signal quality index above 32 were included.

**Fig. 1:**
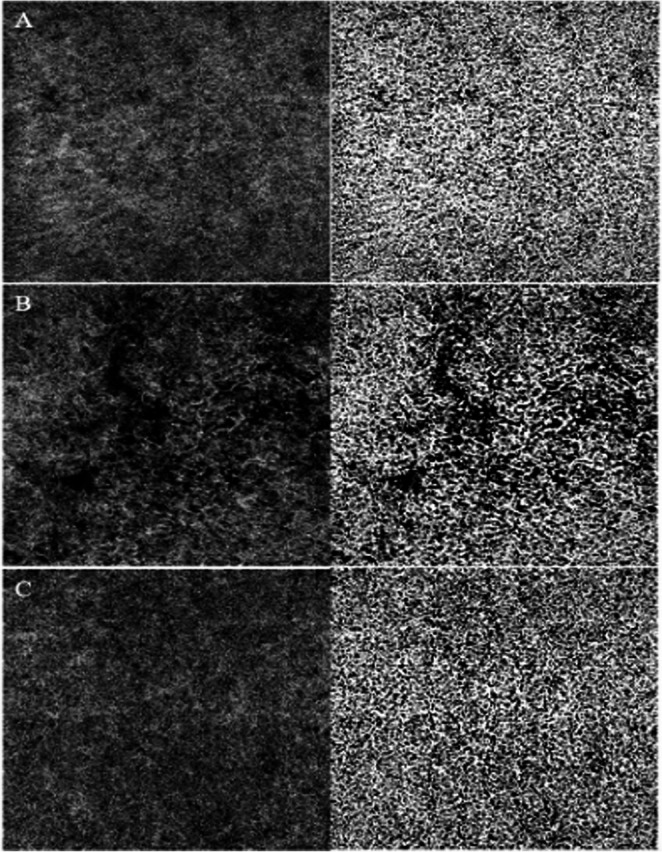
Three en face OCTA images on the left, representative of the choriocapillaris of the fellow eye (**A**), the CSC eye (**B**) and a healthy control eye (**C**), along with the corresponding binarized image on the right

The en-face OCTA images were analyzed using ImageJ software version 1.54 m. To standardize the images, each was resized to 1024 × 1024 pixels. After conversion to 8-bit format, images were binarized using the Phansalkar method with a radius of 15 pixels. Quantitative analysis of choroidal FD was then performed. The ‘Analyze Particles’ command in ImageJ was used to calculate the percentage of flow in each scan relative to the total image area, this process is depicted in Fig. 1. The final FD value was obtained by subtracting the percentage calculated with the ‘Analyze Particles’ command from the total image area.

The FD analysis protocol was adapted from previously published methods for choriocapillaris quantification using OCTA, including the use of local thresholding and particle analysis techniques, as described by Zhang et al. and Byon et al. [16, 23].

### OCT-EDI imaging

A single macular scan was obtained for each eye using the Spectralis device, centered on the central foveal area. These images were captured with a 30° lens in EDI mode, using a horizontal scan and 100 Automated Real Time (ART) frames.

SFCT was calculated using Heidelberg’s “measurement” tool by drawing a perpendicular line from the basal margin of the RPE to the boundary between the choroid and sclera [31].

CVI was calculated using ImageJ, version 1.54 m. The “rectangle” tool was used to delineate the macular area, with the rest cropped. The subfoveal area was selected using the “polygon” tool, bordered by the basal RPE margin and the choroidal-scleral boundary, and was standardized to 750 × 750 μm from the foveal center. This area represents the total choroidal area (TCA).

The total choroidal area was then selected using the “ROI Manager” from the “Analyze” menu in ImageJ. Three choroidal vessels with lumens larger than 100 μm were selected using the “oval” tool, and the average reflectivity of these areas was measured. Mean brightness was adjusted based on the minimum value to reduce background noise using the “Brightness and Contrast” tool.

The image was converted to 8-bit format, binarized using the Niblack method, and converted back to RGB (red, green, blue) format. The luminal area (LCA) was determined using the “Threshold Color” tool, with the range set to values from 0 to 255.

**Fig. 2:**
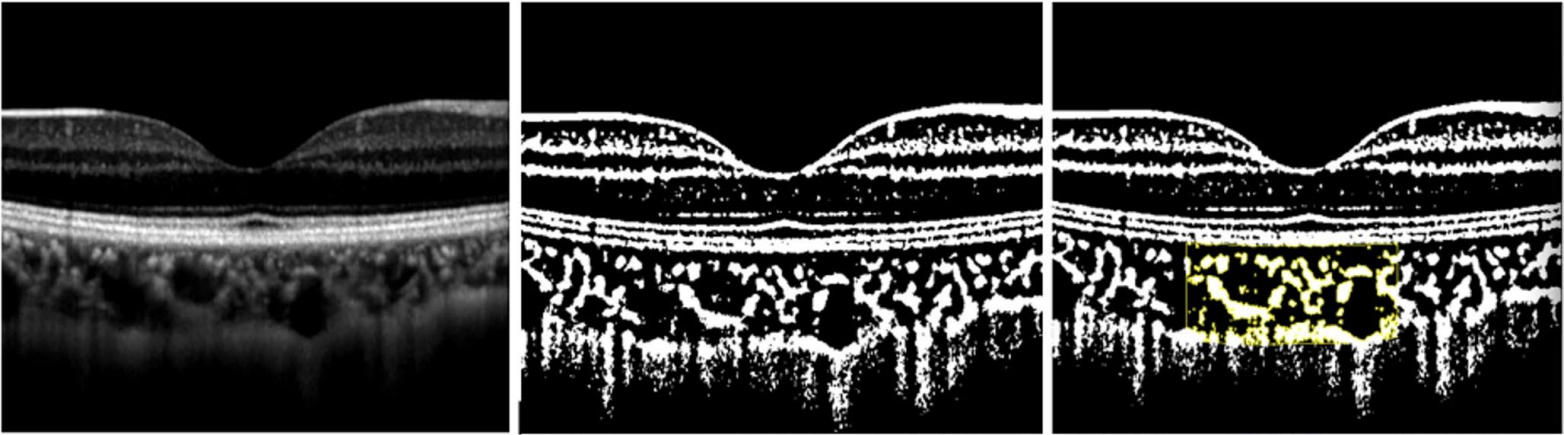
Selected EDI-OCT image (left), then binarized (center), and processed to obtain the luminal area of interest (right image)

The “ROI Manager” was used again to combine the two selected images by pressing “more” to merge them into the choroidal luminal part. Light pixels were defined as the stromal area of the choroid (SCA), and dark pixels as the luminal area (LCA). CVI was calculated as the ratio of LCA to TCA. This analysis process is shown in Fig. 2.

The CVI calculation protocol was based on the binarization method originally described by Sonoda et al. and subsequently validated and refined in multiple studies, using ImageJ software to differentiate luminal and stromal choroidal components [24, 33].

### Statistical analysis

Statistical analysis was performed using SPSS software version 29.0 (latest version, November 13, 2022). The data distribution was assessed using the Shapiro-Wilk test, and group comparisons were made using ANOVA. Pearson’s correlation coefficient (or linear correlation index) was calculated to assess potential linear relationships between the study variables. Statistical significance was set at a p-value less than 0.05.

## Results

This study included 50 eyes from 25 patients with chronic unilateral CSC and 50 eyes from 25 healthy individuals. There were no statistically significant differences in age between patients with unilateral CSC and healthy controls (age *p* < 0.05; 46.7 ± 6.3 vs. 45.1 ± 5.9). 21 patients were male and 4 females in both groups.

The eyes of patients with chronic CSC were divided into two groups: 25 eyes affected by CSC (CSC group) and 25 unaffected fellow eyes (Fellow group).

The healthy group was initially divided into 25 right eyes and 25 left eyes. Since there were no statistically significant differences between right and left healthy eyes (SFCT *p* = 0.88; FD *p* = 0.72; CVI *p* = 0.58), the right eyes of healthy subjects were chosen as the control group.

CSC group had significantly higher mean values of SFCT (320.9 ± 59.5), FD (54.2 ± 9.2), and CVI (52.8 ± 18.2) compared to both the fellow group (SFCT 296.6 ± 51.9; FD 44.5 ± 6.3; CVI 46.5 ± 20.3) and the control group (right eye: SFCT 260.8 ± 71.2; FD 42.3 ± 9.7; CVI 21.5 ± 10.9; left eye: SFCT 263.8 ± 66.6; FD 43.9 ± 9.9; CVI 22.6 ± 11.8).

The Shapiro-Wilk test confirmed the normal distribution of the data.

ANOVA was performed to determine if there were statistically significant differences in the mean values of SFCT, FD, and CVI across the three groups. The results revealed a significant difference between the groups (*p* < 0.05), indicating that the means were not equal.

A post-hoc Bonferroni test was conducted to control for Type I errors due to multiple comparisons and to identify specific group differences. For SFCT, no statistically significant difference was found between the CSC and Fellow groups (*p* = 0.50) and between the Fellow and control group (*p* = 0.13). However, a significant difference was found between the CSC group and the control group (*p* = 0.01).

Considering the CVI, there was no statistically significant difference between the CSC and Fellow groups (*p* = 0.60), but significant statistical differences were found between the CSC and control group (*p* < 0.05) and between the Fellow and control group (*p* < 0.05).

**Fig. 3:**
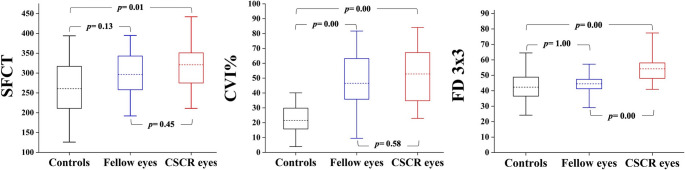
Summary of statistical findings for SFCT, CVI, and FD across all groups

For FD, statistically significant differences were observed between the CSC group and both the Fellow group (*p* < 0.05) and the control group (*p* < 0.05). However, no significant difference was found between the Fellow and control group (*p* = 1.00). The statistical results are reported in Fig. 3.

**Fig. 4:**
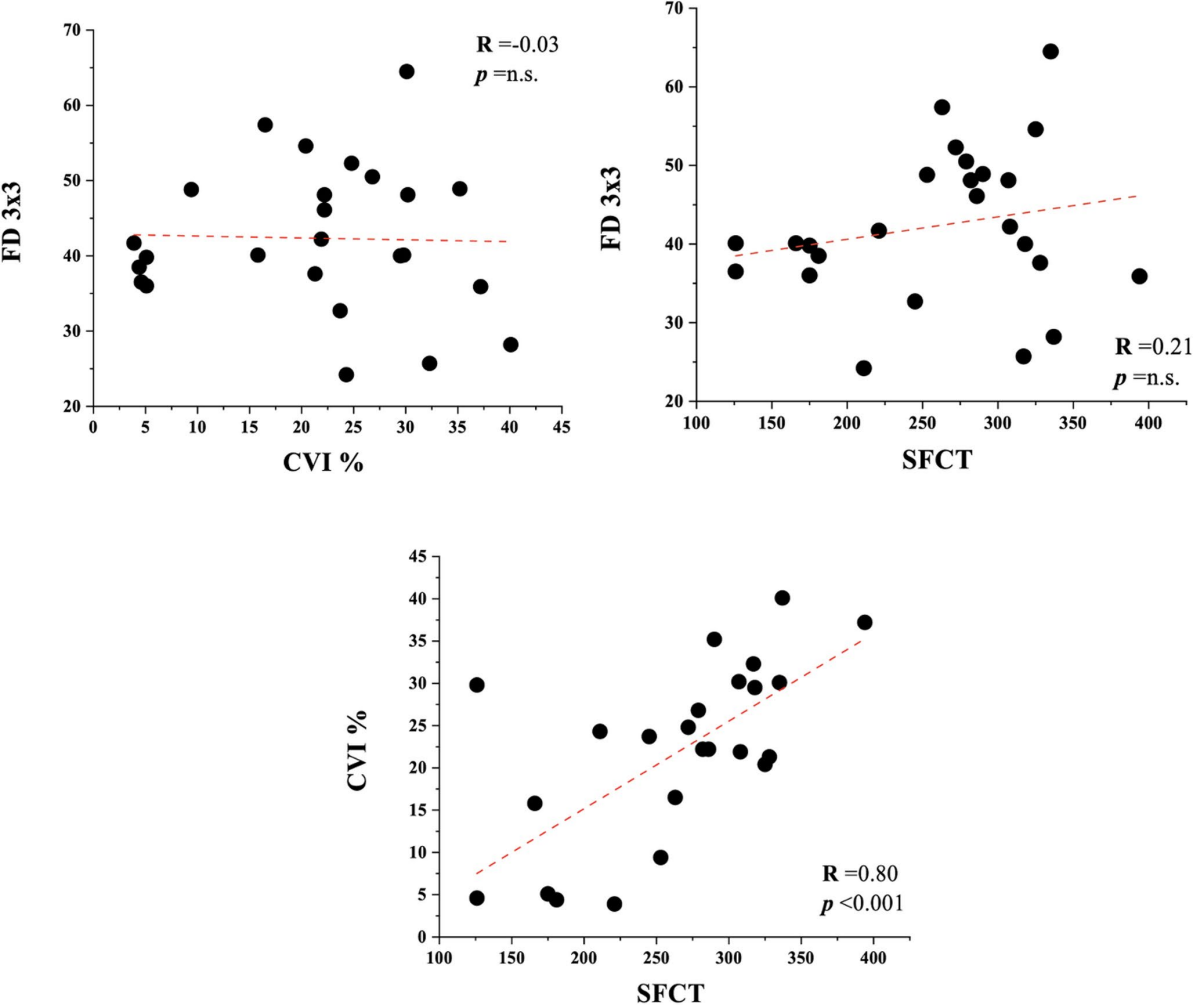
Pearson correlation in the control group

**Fig. 5:**
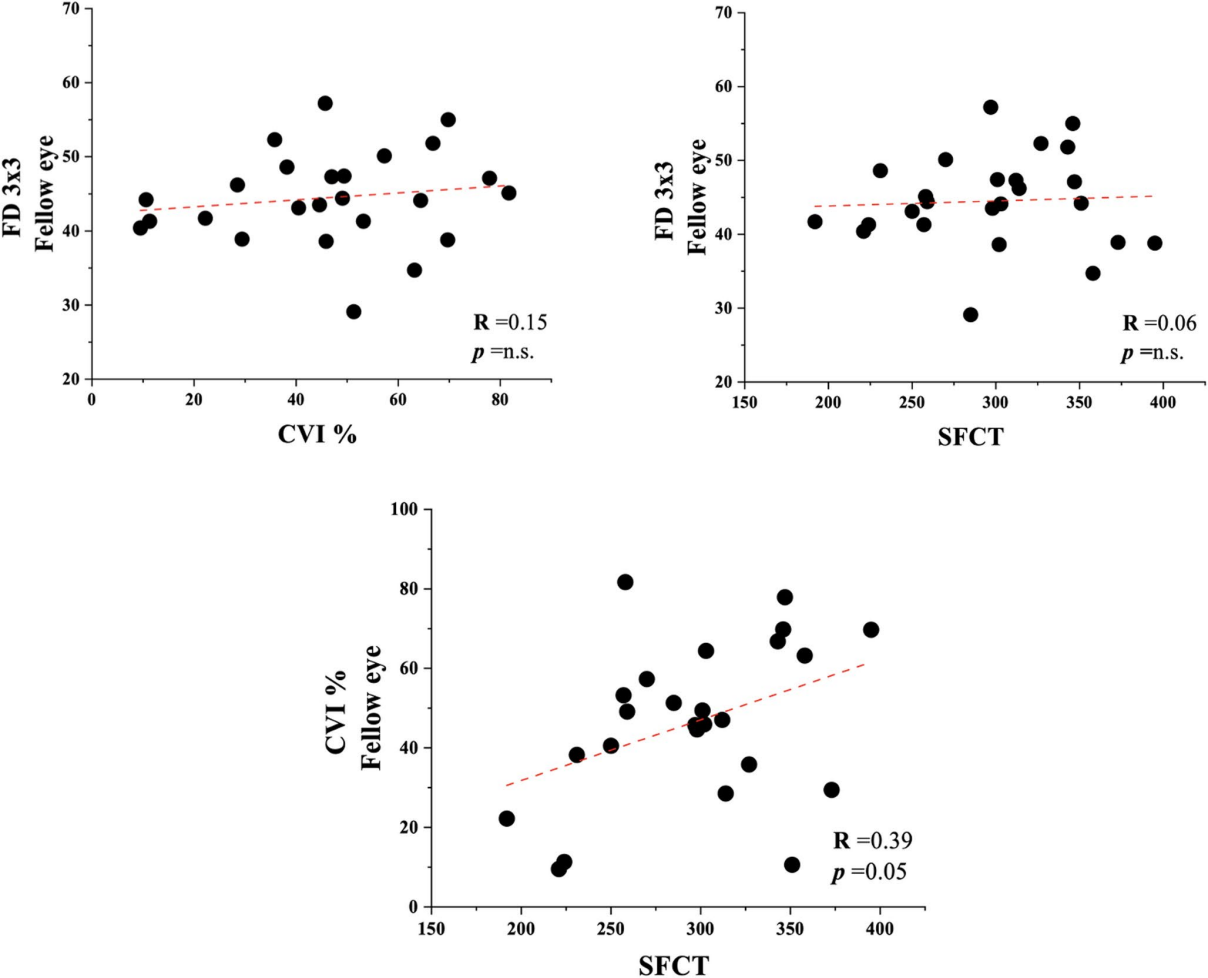
Pearson correlation in the fellow group

**Fig. 6:**
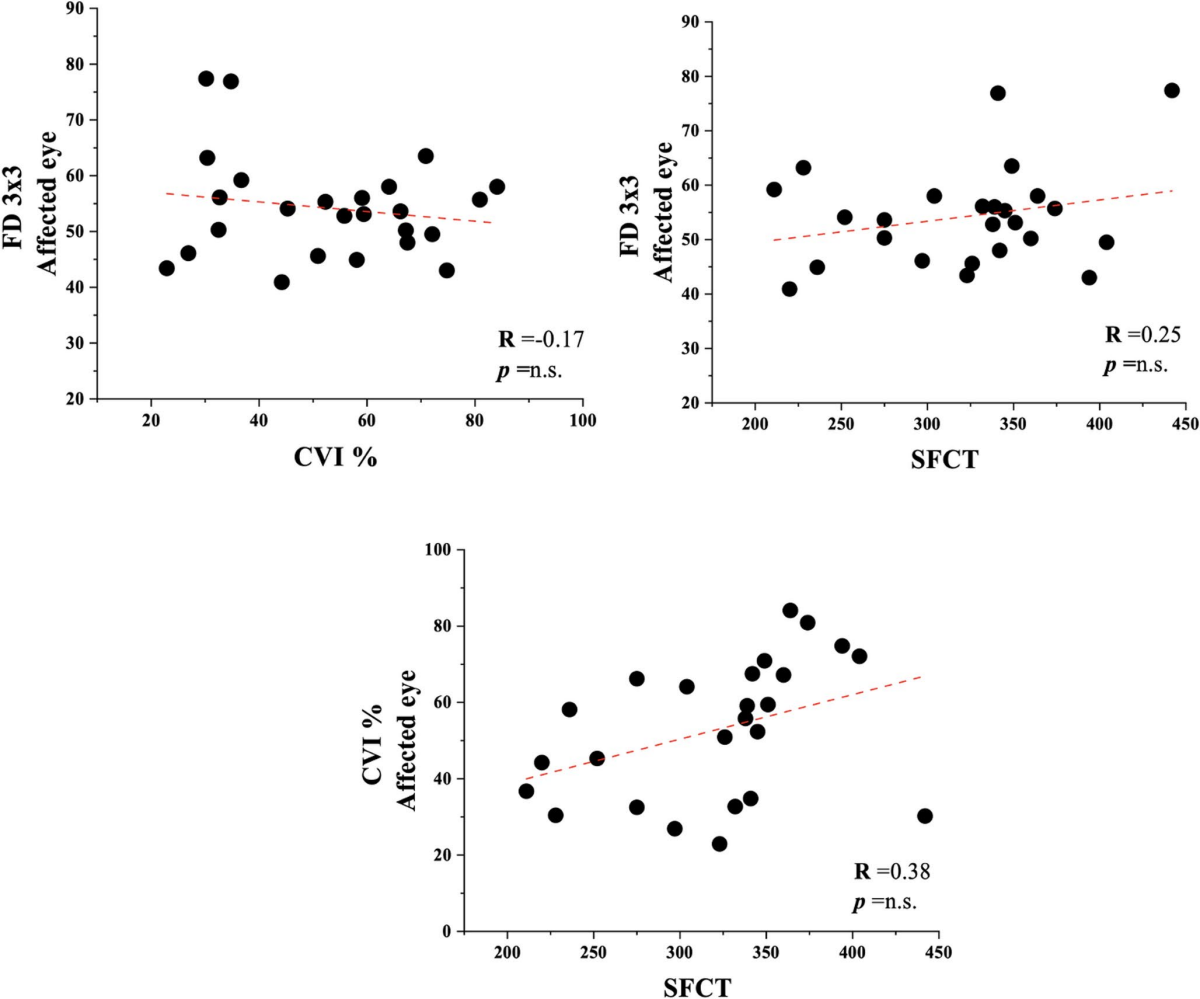
Pearson correlation in the CSC group

Pearson’s correlation coefficient was also used to assess the relationship between parameters. There is always a positive correlation between SFCT and CVI, as well as between SFCT and FD, in both CSC patients and healthy control (R values above 0 indicate a positive linear correlation; R between 0 and 0.3 indicates a weak correlation; R between 0.3 and 0.6, moderate; and R above 0.7, strong). However, there is a negative correlation between FD and CVI in all subgroups except for the fellow eye group, where Pearson’s R shows a weak positive correlation (*R* = 0.15) (Figs. 4, 5, 6).

The statistical significance of the analysis, however, is not always *p* < 0.05, meaning the correlation found is not always reliable. The p-value is statistically significant in the analysis of SFCT and CVI in both the control group (*p* < 0.00) and the Fellow group (*p* = 0.05), but not in the CSC group (*p* > 0.05). In other comparisons, the p-values are consistently not statistically significant, indicating that it cannot be concluded with statistical certainty that the correlation coefficient R is different from zero.

## Discussion

Our findings demonstrate that OCT- and OCTA-derived quantitative parameters reveal measurable macular choroidal and choriocapillaris differences between CSC eyes, fellow eyes, and healthy controls.

While CSC was typically considered a localized condition affecting a single eye, mounting evidence suggests that subclinical alterations may also be present in the fellow eye, pointing to a more systemic or bilateral predisposition [34]. This study aimed to investigate this hypothesis by analyzing key choroidal parameters—SFCT, FD and CVI—in affected eyes, fellow eyes, and healthy controls.

Eyes with CSC exhibit significantly increased SFCT, FD, and CVI compared to healthy controls. In addition, fellow eyes showed intermediate values for these parameters, although not all differences reached statistical significance, indicating the presence of measurable choroidal differences even in the absence of overt clinical manifestations. These results support the concept of bilateral choroidal involvement within the macular area analyzed, rather than implying disease progression or predictive risk.

Results highlighted a significant increase in SFCT in CSC eyes compared to healthy controls, consistent with previous literature indicating that choroidal thickening is a hallmark of CSC.

In our cohort, SFCT did not differ significantly between CSC eyes and fellow eyes, suggesting a limited ability of SFCT alone to discriminate between affected and fellow eyes in chronic unilateral CSC. Increased SFCT in CSC has been reported in multiple studies, supporting the hypothesis that a thickened choroid plays a role in disease pathophysiology [35]. Similarly, a meta-analysis by Chen et al. demonstrated that SFCT is significantly increased in eyes with clinically manifest CSC compared to both fellow eyes and healthy controls [36].

Our study also found that SFCT was elevated in fellow eyes compared to healthy controls, though to a lesser extent than in CSC-affected eyes. This finding is consistent with previous reports, including the meta-analysis by Chen et al. [36]. Within the context of our data, these findings suggest that increased SFCT may represent a shared choroidal characteristic in CSC patients rather than a marker of active disease alone, supporting a bilateral choroidal phenotype.

However, choroidal thickness does not fully account for underlying vascular changes; in this context, the CVI has been proposed as a more specific marker of choroidal vascular involvement. Accordingly, Agarwal et al. reported a significantly increased CVI in CSC-affected eyes compared to healthy controls, suggesting that vascular congestion plays a key role in disease pathogenesis [37].

A meta-analysis by Xia et al. further reinforced these findings, showing higher CVI in both CSC and fellow eyes, supporting the idea of a bilateral choroidal involvement even in clinically unaffected eyes [27].

However, discrepancies exist in the literature regarding the extent and significance of CVI changes in fellow eyes, and methodological differences—such as the area selected for CVI calculation—may influence results [27].

In our study, CVI values were significantly higher in CSC eyes compared to healthy controls, while fellow eyes exhibited intermediate values, supporting the presence of subclinical vascular alterations. Similar findings were reported by Sahoo et al., who demonstrated altered CVI in CSC eyes and milder changes in fellow eyes, along with weaker correlations between CVI and choroidal thickness in fellow eyes [38]. Taken together, these results suggest that CVI alterations may extend beyond the clinically affected eye, although their magnitude and clinical significance appear reduced in fellow eyes.

Regarding choriocapillaris flow, our study confirmed an increase in FD in CSC eyes, indicating reduced choriocapillaris perfusion within the analyzed macular region. These findings reflect localized alterations in choriocapillaris flow rather than a direct assessment of deeper choroidal vascular structures.

Current evidence suggests that choroidal vascular hyperpermeability in CSC is primarily driven by dilated pachyvessels and pachyvenous anastomoses, rather than by a primary alteration of the choriocapillaris [39].

In this context, the increased choriocapillaris FD observed in CSC and fellow eyes are more likely to represent secondary ischemic changes caused by mechanical compression or displacement of the inner choroid by enlarged outer choroidal vessels. This interpretation supports the concept of choriocapillaris involvement as collateral damage within the pachychoroid spectrum, rather than as the initial pathogenic event [39–41].

Importantly, our study also observed a mild increase in FD in fellow eyes compared to healthy controls. Although no longitudinal or prospective conclusions can be drawn, these findings are consistent with previous OCT-A studies reporting choriocapillaris flow alterations in fellow eyes of patients with unilateral CSC [41, 42]. Within the limits of a cross-sectional design, our results indicate the presence of measurable macular choriocapillaris changes in fellow eyes, without implying temporal progression or causality.

A potential limitation of this study is the relatively narrow age range of the study population. The age range of 30 to 55 years was intentionally selected to reduce the potential confounding effects of age-related changes in choroidal thickness, choroidal vascularity, and choriocapillaris perfusion, which are known to influence OCT- and OCTA-derived measurements [22]. While this choice strengthens the internal validity of the comparisons across study groups, it may limit the generalizability of the findings to younger or older populations. A further potential limitation of this study is the use of a SD-OCTA device rather than a swept-source OCT (SS-OCT) system. SS-OCT offers deeper tissue penetration and may provide advantages in the evaluation of choriocapillaris flow, particularly in eyes with increased choroidal thickness [43]. Nevertheless, SD-OCT and SD-OCTA, remains a validated and widely used imaging modality in CSC research for the analysis of choriocapillaris flow deficits [44].

While these quantitative imaging findings improve the pathophysiological characterization of CSC, their direct therapeutic implications remain to be defined. At present, OCT- and OCTA-derived parameters primarily contribute to disease understanding and phenotyping rather than guiding specific treatment decisions, highlighting the need for future prospective and interventional studies.

In conclusion, our study demonstrates the presence of measurable macular choroidal and choriocapillaris differences in CSC and fellow eyes based on OCT and OCTA-derived parameters. The significant alterations observed in CSC eyes and the intermediate values detected in fellow eyes suggest a bilateral choroidal phenotype confined to the analyzed imaging area. These findings underscore the importance of interpreting OCT- and OCTA-derived parameters strictly in relation to the acquired data, while avoiding extrapolation beyond the imaging field and the study design.
